# Vitamin D, Folate and the Intracranial Volume in Schizophrenia and Bipolar Disorder and Healthy Controls

**DOI:** 10.1038/s41598-018-29141-y

**Published:** 2018-07-17

**Authors:** Tiril P. Gurholt, Kåre Osnes, Mari Nerhus, Kjetil N. Jørgensen, Vera Lonning, Akiah O. Berg, Ole A. Andreassen, Ingrid Melle, Ingrid Agartz

**Affiliations:** 1 Norwegian Centre for Mental Disorders Research (NORMENT), KG Jebsen Centre for Psychosis Research, Division of Mental Health and Addiction, Institute of Clinical Medicine, University of Oslo, Oslo, Norway; 2 Department of Psychiatric Research, Diakonhjemmet Hospital, Oslo, Norway; 3 Division of Mental Health, Akershus University Hospital, Lørenskog, Norway; 4 Division of Mental Health and Addiction, Oslo University Hospital, Oslo, Norway; 5 Department of Clinical Neuroscience, Centre for Psychiatric Research, Karolinska Institutet, Stockholm, Sweden

## Abstract

Vitamin D and folate deficiency are considered risk factors for schizophrenia and bipolar disorders, but it is unknown how vitamin D and folate influence the growing brain, cranium or the clinical phenotype. Serum vitamin D and folate levels are in part genetically regulated. We investigated whether adult vitamin D and folate levels are associated with the intracranial volume (ICV) under the hypothesis that developmental vitamin D or folate levels influence neurodevelopment and that current levels are associated with ICV. Ninety patients with severe mental disorders and 91 healthy controls underwent 3 T magnetic resonance imaging and serum sampling. Multiple linear regression was used to assess the contribution of serum vitamin D, folate and patient-control status on ICV. We show that vitamin D levels were within lower range for patients and controls (48.8 ± 22.1 nmol/l and 53.4 ± 20.0 nmol/l, respectively). A significant positive association was found between vitamin D and ICV (p = 0.003, r = 0.22), folate was trend-significantly associated with ICV. Folate and vitamin D were significantly associated (p = 0.0001, r = 0.28). There were nonsignificant patient-control differences and no interaction effects. The results suggest that Vitamin D is associated with ICV as detected in the adult. Further studies are warranted for replication and to investigate possible mechanisms and genetic associations.

## Introduction

The size of the intracranial volume (ICV) depends on the developing brain and is considered fully grown by early adolescence^1^. Severe mental disorders such as schizophrenia and bipolar disorders have been hypothesized to be part of a neurodevelopmental continuum^2^. Recent meta-analyses indicate significantly smaller ICV in schizophrenia when compared to controls^3,4^, but this has not been demonstrated for bipolar disorder^5^. Smaller ICV in schizophrenia could suggest abnormal brain development prenatally, in childhood or in early adolescence, i.e. prior to psychosis onset in the majority of patients^1^. Although the causal reason for smaller ICV is unknown, one hypothesis relates it to developmental nutritional deficiencies during gestation, childhood or adolescence. A small study supports this notion by indicating smaller ICV in schizophrenia patients exposed to prenatal famine^6^. There is less evidence of aberrant neurodevelopment for bipolar disorder.

Schizophrenia and bipolar disorder have been suggested to be part of a heterogeneous schizophrenia-bipolar spectrum^7^. Epidemiological studies indicate that developmental nutritional deficiencies e.g. due to maternal famine or winter-spring births are associated with increased risk for offspring with schizophrenia^8,9^ and bipolar disorder^10,11^. This was supported by a Swedish register study where inadequate gestational weight gain, a proxy for inadequate maternal nutrition, was associated with higher risk for offspring with non-affective psychosis^12^. Developmental serum vitamin D and serum folate (S-folate) deficiencies are considered risk factors for the development of schizophrenia^8^. Results from a Danish register study supports this by indicating a nonlinear relation between neonatal vitamin D levels and schizophrenia risk^13^, while a trend level increase in schizophrenia risk for offspring with dark-skin in the presence of maternal vitamin D deficiency was reported in a study from Boston, USA^14^. A birth cohort study from California, USA, reported that elevated maternal homocysteine was associated with increased risk for offspring schizophrenia^15^. Elevated homocysteine is a marker for folate deficiency^8^. These findings indicate that prenatal nutritional deficiencies could predispose the offspring for schizophrenia with implications for brain and cranial development, while there is a need for studies in schizophrenia-bipolar spectrum disorders.

Vitamin D is a secosteroid hormone that is obtained from the diet, diet supplementation, or synthetized naturally from sunshine^16^. Its main circulating form is 25-hydroxyvitamin D (S-25(OH)D) which in its active form, 1,25-dihydroxyvitamin D, binds to vitamin D receptors^16^ that are found in cells throughout the body^16^, including the human brain^17^. Vitamin D deficiency has been defined as levels of S-25(OH)D < 25, insufficiency as 25 ≤ S-25(OH)D < 50, suboptimal as 50 ≤ S-25(OH)D < 75, and sufficiency as S-25(OH)D ≥ 75 in nmol/l^18,19^. Vitamin D deficiency is traditionally associated with bone disease although newer research also indicates its importance for other disease outcomes^19^. Animal studies show that developmental vitamin D deficiency influence brain development and brain gene expression^20,21^. In humans, previous studies report an inverse association between S-25(OH)D and ICV in adults^22,23^. Thus, ICV could be influenced by current micronutrient status or their genetic regulation in adults. It is unknown if the same associations are found in schizophrenia and bipolar spectrum disorders.

Folate is an essential micronutrient obtained from the diet or from diet supplementation^8^, and is also synthetized locally by the intestinal microbiome^24^. The methylenetetrahydrofolate reductase (MTHFR) enzyme, influenced by the MTHFR gene, produces the main form of circulating folate (S-folate)^25^ and folate receptors are found in the human brain^26,27^. The S-folate deficiency cutoff depends on the laboratory reference value, but it is commonly 7–10 nmol/l. Animal studies indicate that maternal MTHFR^28^ or folate^29,30^ deficiency, as well paternal folate deficiency^31^, have adverse effect on the developing fetus that include brain changes. In humans, there is inconclusive evidence of how MTHFR polymorphism confers with the risk of schizophrenia^9^ or bipolar disorder^32^, though a meta-analysis reported MTHFR polymorphism to be associated with schizophrenia, bipolar and major depression disorders when analyzed together^33^. There are indications that elevated maternal homocysteine increase the risk of schizophrenia^15^. To our knowledge, no study has assessed whether there is an association between S-folate and ICV.

Ethnicity could be a factor in clinical studies. More Vitamin D synthetization exposure is needed for darker pigmentations^34^. S-25(OH)D is heritable and influenced by genetic variations^35,36^, as is S-folate^37,38^. ICV also has a genetic component^39^ that overlap with height genes^40^. Ethniticy is a known confounding variable in genetic studies, and potentially also for studies of micronutrient and brain structure.

In the scientific literature there are indications that lifestyle and socioeconomic factors could influence brain structure. Obesity has been associated with negative structural brain changes in adolescence and in adulthood^41^, and weight gain was indicated to have negative influence on brain structure in both adult psychosis patients and controls^42^. Recently, obesity was found associated with higher brain age in first-episode psychosis patients^43^ and a meta-analysis linked obesity to cognitive impairment in schizophrenia^44^. Socioeconomic factors were also recently indicated to influence brain structure in children and adolescence, and lower socioeconomic status was associated with negative effects, although this could coincide with poorer nutrition and less stimulating childhood environment^45^. Furthermore, a Danish register study recently indicated that schizophrenia risk was associated with lower parental socioeconomic status, a family history of psychosis and polygenic risk score, with possibly unrelated genetic and socioeconomic pathways^46^. These and other environmental factors have the potential to influence brain structures, including ICV as measured in adults, in addition to nutritional factors.

Folate and vitamin D levels have both been implicated as risk factors for developing schizophrenia and bipolar spectrum disorders, as well as in brain and bone development. Vitamin D is essential for cranial development in early life, and vitamin D and folate insufficiency could be associated with the smaller ICV as reported in schizophrenia. The aim of the present study was to investigate a putative association between current vitamin D and S-folate levels with ICV measured from magnetic resonance imaging (MRI) scans of adult patients with severe mental disorders and healthy controls. We expected to find vitamin D and S-folate to be associated with ICV both in the patients with severe mental disorders and the healthy controls, but with a stronger association in the patients. We expected vitamin D to have a stronger association with ICV than S-folate.

## Methods and Materials

### Study population

The subject sample consisted of 90 patients with severe mental disorders (*Schizophrenia spectrum disorder* [N = 34] (Schizophrenia [N = 24], Schizophreniform [N = 1], Schizoaffective disorder [N = 9]), *Bipolar disorder* [N = 40] (Bipolar I [N = 24], Bipolar II [N = 13], Bipolar NOS [N = 3]), *other psychoses* [N = 16] (Major depressive disorder with psychosis [N = 2], other psychotic disorders [N = 14]) and 91 healthy controls. Patients were recruited from psychiatric hospitals and outpatient clinics in the Oslo region, Norway, as part of the ongoing Thematically Organized Psychosis (TOP) Research Study. Healthy controls were recruited from the same geographic area by random draw from the national population registry and invited by letter to participate. They were screened for current and previous psychiatric disorders and excluded if they had experienced a psychiatric disorder. Participants were included from all ethnic backgrounds, but the controls were of mostly Caucasian origin (see Table 1). Exclusion criteria for all participants were: age <18 or >65 years, IQ <70, previous moderate or severe head injury or a neurologic illness. Participants were also excluded if they did not have height and weight measurements. All participants received oral and written information about the study and signed a written informed consent. The study was approved by the Regional Committee for Medical Research Ethics and the Norwegian Data Inspectorate and was conducted in accordance with the Helsinki declaration.

### Clinical Assessment

Diagnoses were assessed by trained psychologists or physicians using the Structured Clinical Interview for DSM-IV axis I disorders (SCID-I) modules A–E^47^. Psychosocial functioning was assessed using the split version of the Global Assessment of Function (GAF-S and GAF-F) scale^48^. Age at onset was defined as the age of first SCID-I verified positive psychotic symptom experience or for bipolar disorder patients the first manic, hypomanic or major depressive episode. Duration of illness was calculated from age at onset to age at MRI.

### Serum measurements

The serum sample resides in room temperature for at least 30 minutes and maximally 2 hours prior to being centrifuged and blood cells and serum separated at which time the samples were refrigerated. They were transferred for S-25(OH)D and S-folate measurement the same day. Total S-25(OH)D was determined by a liquid chromatography-tandem mass spectrometry method^49^ and S-folate by electro-chemiluminescence immunoassay (Roche Diagnostics, Mannheim, Germany) with laboratory reference value of S-folate < 10 nmol/l.

The inclusion was limited to subjects with S-25(OH)D and S-folate measurements within six weeks of MRI. The maximum cross season (winter: November-April, summer: May-October) discrepancy was three weeks.

### Data acquisition

Patients and controls underwent MRI on the same 3.0 T General Electric (GE) Healthcare Signa HDxt MR scanner (GE Medical Systems, Milwaukee, Wisconsin, USA) equipped with an 8-channel head coil. After running 3D localizer and axial asset calibration, one sagittal T1-weighted fast spoiled gradient echo (FSPGR) sequence was acquired with echo time [TE] = Min Full, repetition time [TR] = 7.8 s, inversion time [TI] = 450 ms, flip angle = 12°, field of view [FOV] = 256 mm and voxel size = 1 × 1 × 1.2 mm^3^. There was no major scanner upgrade or change of instrument during the study period.

### MRI Processing

All MRI scans were processed using FreeSurfer^50^ version 5.3.0. Briefly, the processing steps include motion correction, bias field correction, brain extraction, intensity normalization and automatic Talairach transformation, with optimized 3 T bias field filtration^51^. Estimated ICV^52^ was obtained through the subcortical segmentation stream^53^. The segmentation quality was assessed by manual inspection of outlier volumes (defined as: ≥1.5 times interquartile range of key subcortical, total cortex and cerebellum cortex volumes) and excluded if the segmentation was inaccurate.

### Statistical method

The demographic variables of patients and controls were compared using two-sample t-test for continuous variables with a normal distribution and the two-sided Wilcoxon rank sum test was used for continuous variables with a non-normal distribution. The distributions were considered normal if they passed the Shapiro-Wilk’s normality test^54^ and their histograms were normal from visual evaluation. For categorical variables the χ^2^ **-**test was used.

Multiple linear regression was used to investigate the association between S-25(OH)D, S-folate and ICV, and between S-25(OH)D and S-folate, using the R statistical software’s *lm* function. The association between S-25(OH)D, S-folate and ICV was investigated both individually (Model 1 and Model 2, respectively) and together (Model 3) while also adjusting for group (patient-control status), age, sex, weight, height, ethnicity (Caucasian or non-Caucasian origin) and assessment season (winter: November-April, summer: May-October). Post hoc analysis were performed in post hoc Model A and Model B to investigate the association between S-25(OH)D and S-folate, firstly while adjusting for group, age, sex, ethnicity and assessment season, and secondly while also adjusting for weight and height. In the supplemental information, additional post hoc analyses explored the possibility of group and season specific contributions on the above models. Group, ethnicity and assessment season are binary variables.

The regression results are expressed as standardized β coefficients, 95% confidence interval and p-values. The effect size is reported as Cohen’s d for categorical variables and the partial correlation coefficient, r, for continuous variables^55^. F statistics was used to assess the contribution of additional covariates^56^.

### Data availability

The analysis script is available upon request to the first author. Clinical data are not publicly available and cannot be shared.

## Results

### Demographic, Clinical Data and Imaging Information

Table 1 presents the demographic, clinical variables and MRI information. Patients and healthy controls are similar in numbers, sex, age and S-folate levels, while there is a trend-significant difference for S-25(OH)D (p = 0.094). On average, patients have insufficient and controls suboptimal S-25(OH)D levels, while both patients and controls have normal S-folate levels. The patients and controls are also similar with respect to weight and body mass index (BMI), although there is a trend towards patients being shorter than controls (p = 0.078). Patients have significantly less education than controls (p = 0.0001) with an average of 13.3 ± 2.3 (mean ± standard deviation) years of education compared to 14.7 ± 2.5 years of education in controls. The majority of the patients’ parents have higher education, this information was not available for the controls. Patients also have significantly smaller average ICV (p = 0.008) (unadjusted for sex and height) than controls. Ethnicity differ significantly between the groups (p = 3.6e-06); 25.6% of patients and 1.1% of controls are of non-Caucasian origin. The average number of days between MRI and blood sampling are 7.9 ± 8.2 for patients and 14.6 ± 11.5 for controls. The clinical measures indicated that patients are, on average, in a fairly stable-symptomatic state. In Supplementary Note 1, Table S1, the influence of ethnicity on the demographic, clinical and MRI information is presented for the patients.

**Table 1:** Demographic and clinical characteristics for patients with severe mental disorders and healthy controls.

	Patients (N = 90)	HC (N = 91)	Two-sample t-test, Wilcoxon rank sum test or χ^2^	p-value
**Clinical information** ^a^				
Women, N (%)	47 (52.2)	37 (40.7)	1.99	0.158
Age (years)^b^	30.6 (10.8)	31.5 (8.2)	−1.56	0.118
Non-Caucasians, N (%)	23 (25.6)	1 (1.1)	21.45	**3.6e-06**
Assessed summer, N (%)	40 (44.4)	49 (53.9)	1.25	0.264
Education (years)^b,c^	13.3 (2.3)	14.7 (2.5)	−3.89	**0.0001**
Mother college educated, N (%)^d^	48 (57.1)			
Father college educated, N (%)^d^	45 (55.6)			
Height (cm)	174.3 (9.0)	176.7 (9.2)	−1.78	0.078
Weight (kg)^b^	78.5 (18.1)	76.1 (13.4)	0.49	0.628
BMI^b^	25.7 (5.1)	24.3 (3.3)	1.64	0.100
AAO (years)	22.2 (8.3)			
DOI at MRI (years)	8.4 (8.4)			
**Clinical measurements**				
GAF-F	51.3 (12.2)			
GAF-S	52.4 (13.3)			
Total PANSS score	49.7 (12.4)			
Negative PANSS score	11.6 (4.3)			
Positive PANSS score	11.2 (4.3)			
**Serum measurements**				
S-25(OH)D (nmol/l)^b^	48.8 (22.1)	53.4 (20.0)	−1.68	0.094
S-folate (nmol/l)^b^	20.6 (9.6)	19.2 (7.4)	0.56	0.573
**Imaging information**				
Estimate ICV (ml)^b^	1467.6 (129.1)	1512.4 (127.9)	−2.67	**0.008**

*Notes:* Significance threshold p < 0.05 indicated in bold. For continuous variables, two-sample t-test applied for normal data, otherwise two-sided Wilcoxon rank sum test. χ^2^-test is applied for categorical data. Patients: patients with severe mental disorders, HC: healthy controls, MRI: magnetic resonance imaging, winter: November-April, summer: May-October, Ethnicity: Caucasian or non-Caucasian origin, BMI: Body-Mass Index (weight in kg/meter squared), AAO: Age at onset, DOI: Duration of Illness, GAF: Global Assessment of Functioning, GAF-F: GAF–function scale, GAF-S: GAF-symptom scale, PANSS: Positive and Negative Syndrome Scale, S-25(OH)D: 25-hydroxyvitamin D, ICV: Intracranial Volume.

^a^Presented as mean (standard deviation), if not stated otherwise.

^b^Data non-normally distributed.

^c^Computed based on data from 78 (86.7%) patients and 90 (98.9%) controls.

^d^Computation based on data from 84 (93.3%) mothers and 81 (90.0%) fathers.

### S-25(OH)D and S-folate’s association with ICV

Table 2 summarizes the association between S-25(OH)D, S-folate and ICV in patients and controls. In Model 1, there is a significant positive association for S-25(OH)D on ICV (p = 0.003, r = 0.22) after adjusting for group (patient-control status), age, weight, height, sex, ethnicity and assessment season. There are also significant negative associations between ICV and weight (p = 0.004, r = −0.22), and significant positive associations for ICV and height (p = 4.5e-05, r = 0.30) and between ICV and sex (p = 6.4e-06, d = 0.71). Non-Caucasians origin was associated with smaller ICV (p = 0.041, d = −0.31). However, note that non-Caucasian origin here coincide with patient status with 95.8% of non-Caucasians being patients. ICV is not significantly associated with patient-control status, age or assessment season. Model 1 has adjusted R^2^ = 0.41.

**Table 2:** The association between S-25(OH)D, S-folate and ICV for patients and controls.

Covariates	Dependent variable: ICV								
	Model 1			Model 2			Model 3		
	β	95% CI	p-value	β	95% CI	p-value	β	95% CI	p-value
S-25(OH)D	0.2	(0.1, 0.3)	**0.003**				0.2	(0.04, 0.3)	**0.011**
S-folate				0.1	(−0.002, 0.2)	0.053	0.1	(−0.1, 0.2)	0.233
Group^a^	0.01	(−0.2, 0.3)	0.92	−0.02	(−0.3, 0.2)	0.856	−0.01	(−0.3, 0.2)	0.929
Age	−0.001	(−0.1, 0.1)	0.99	−0.003	(−0.1, 0.1)	0.968	−0.02	(−0.1, 0.1)	0.799
Sex^b^	0.8	(0.5, 1.2)	**6.4e-06**	0.8	(0.5, 1.2)	**1.2e-05**	0.8	(0.5, 1.2)	**5.1e-06**
A. season^c^	−0.04	(−0.3, 0.2)	0.72	0.1	(−0.2, 0.3)	0.688	−0.03	(−0.3, 0.2)	0.794
Weight	−0.2	(−0.4, −0.1)	**0.004**	−0.2	(−0.4, −0.1)	**0.007**	−0.2	(−0.4, −0.1)	**0.007**
Height	0.4	(0.2, 0.6)	**4.5e-05**	0.4	(0.2, 0.6)	**5.5e-05**	0.4	(0.2, 0.6)	**5.4e-05**
Ethnicity^d^	−0.4	(−0.8, −0.02)	**0.041**	−0.5	(−0.9, −0.1)	**0.017**	−0.4	(−0.8, 0.01)	0.054

*Notes:* Regression model with N = 181 participants (90 patients, 91 controls) and standardized continuous covariates. Model 1: S-25(OH)D on ICV, Model 2: S-folate on ICV, Model 3: S-25(OH)D and S-folate on ICV. Significance threshold p < 0.05 indicated in bold. Group: Patient-Control status, CI: Confidence Interval, winter: November-April, summer: May-October, Ethnicity: Caucasian or non-Caucasian origin, A. season: Assessment season.

^a^Reference group: controls.

^b^Reference sex: women.

^c^Reference season: winter.

^d^Reference ethnicity: Caucasian.

In Model 2, the association between S-folate and ICV is assessed while also adjusting for group (patient-control status), age, weight, height, sex, ethnicity and assessment season. There, a trend-significant positive association between S-folate and ICV (p = 0.053, r = 0.15) is observed. As in Model 1, there is a negative weight (p = 0.007, r = −0.21), and positive height (p = 5.5e-05, r = 0.30) and sex (p = 1.2e-05, d = 0.69) associations with ICV that are significant. In addition, non-Caucasian origin is associated with ICV (p = 0.017, d = −0.34). Note that, as previously stated, this could be a patient effect. ICV is, as in Model 1, not significantly associated with patient-control status, age and assessment season. Model 2 explains less variance than Model 1 with adjusted R^2^ = 0.39.

Model 3 assess both the association between S-25(OH)D and S-folate with ICV while also adjusting for the before mentioned covariates. There S-25(OH)D is significantly associated with ICV (p = 0.011, r = 0.19) as before, while S-folate is not significantly associated with ICV. Weight (p = 0.007, r = −0.20), height (p = 5.4e-05, r = 0.30) and sex (p = 5.1e-06, d = 0.72) are significantly associated with ICV as before, while group, age and assessment season are non-significantly associated with ICV. Ethnicity is not significantly associated with ICV after simultaneously adjusting for both S-folate and S-25(OH)D. Model 3 has, similarly to Model 1, adjusted R^2^ = 0.41. F statistics indicate that for Model 1 the inclusion of S-folate as a covariate in Model 3 yields a nonsignificant contribution (F = 1.43, p = 0.233), while for Model 2 the inclusion of S-25(OH)D in Model 3 yields a significant contribution (F = 6.62, p = 0.011) (see also Supplementary Table S4).

In Table 3, post hoc analyses are performed to assess the association between S-25(OH)D and S-folate. In post hoc Model A the association is assessed while also adjusting for patient-control status, age, sex, assessment season and ethnicity, while weight and height are additionally included as covariates in post hoc Model B. In Model A it is shown that S-folate is significantly associated with S-25(OH)D (p = 0.0001, r = 0.28), as is assessment season (p = 0.0005, d = 0.54) with lower measures during winter. Ethnicity is significantly associated with S-25(OH)D (p = 0.011, d = −0.39), indicating lower S-25(OH)D levels in non-Caucasian than Caucasian participants. Patient-control status, sex and age are not significantly associated with S-25(OH)D. Post hoc model A has adjusted R^2^ = 0.20. In post hoc Model B similar results are obtained when also adjusting for weight and height, although adjusted R^2^ = 0.19 which is lower than for Model A. F statistics indicates that the inclusion of weight and height as covariates in post hoc Model B yields a non-significant contribution when compared to post hoc Model A (F = 0.12, p = 0.889). Furthermore, this indicates that the association between S-25(OH)D and ICV in Models 1 and 3 is not driven by an association between S-25(OH)D and weight or S-25(OH)D and height.

**Table 3:** The association between S-folate and S-25(OH)D for patients and controls.

Covariates	Dependent variable: S-25(OH)D					
	Post hoc Model A			Post hoc Model B		
	β	95% CI	p-value	β	95% CI	p-value
S-Folate	0.3	(0.1, 0.4)	**0.0001**	0.3	(0.1, 0.4)	**0.0002**
Group^a^	−0.1	(−0.4, 0.2)	0.555	−0.1	(−0.4, 0.2)	0.630
Age	0.1	(−0.1, 0.2)	0.308	0.1	(−0.1, 0.2)	0.274
Sex^b^	−0.1	(−0.4, 0.1)	0.327	−0.1	(−0.6, 0.3)	0.478
A. season^c^	0.5	(0.2, 0.7)	**0.0005**	0.5	(0.2, 0.7)	**0.0007**
Weight				−0.04	(−0.2, 0.1)	0.651
Height				0.03	(−0.2, 0.2)	0.753
Ethnicity^d^	−0.6	(−1.0, −0.1)	**0.011**	−0.6	(−1.0, −0.1)	**0.014**

*Notes:* Regression model with N = 181 participants (90 patients, 91 controls) and standardized continuous covariates. Post hoc Model A: S-folate on S-25(OH)D, Post hoc Model B extends Model A and includes weight and height as covariates. Significance threshold p < 0.05 indicated in bold. Group: Patient-Control status, winter: November-April, summer: May-October, Ethnicity: Caucasian or non-Caucasian origin, CI: Confidence Interval, A. season: Assessment season.

^a^Reference group: controls.

^b^Reference sex: women.

^c^Reference season: winter.

^d^Reference ethnicity: Caucasian.

In Supplementary Note 2, Tables S2–S5, additional post hoc analyses are performed to further investigate the observed results. The analyses do not indicate any patient specific associations on ICV nor on S-25(OH)D. Furthermore, given the significant contribution of assessment season on S-25(OH)D in Table 3, we investigate the potential of a season specific association between the serum measures and ICV and between assessment season and S-folate on S-25(OH)D. We do not observe any season specific associations.

## Discussion

The main finding of this study was that S-25(OH)D was positively associated with ICV in patients with severe mental disorders and in healthy controls. There was also a positive trend-significant association between S-folate and ICV in patients and controls, but when including both S-25(OH)D and S-folate in the same model, only S-25(OH)D was significantly associated with ICV. For both patients and controls, height was positively associated with ICV as expected due to the genetic overlap^40^ and there were expected sex differences (women < men). In line with a recent meta-analysis in bipolar disorder^5^, but contrary to the results from meta-analyses for schizophrenia^3,4^, the patients were not found to have smaller ICV than healthy controls. There were no interaction effects.

Further investigations showed a significant positive association between S-25(OH)D and S-folate, and there was also an expected significant increase in S-25(OH)D levels during summer. Non-Caucasian ethnicity was significantly associated with having lower S-25(OH)D as previously indicated in reports from Oslo, Norway^49,57^. The association between S-25(OH)D and S-folate is interpreted to explain the trending association between S-folate and ICV, although based on the results it cannot be ruled out that S-folate has an independent association with ICV.

This is, to our knowledge, the first study to report independent and positive associations of S-25(OH)D on ICV in both patients with severe mental disorders and healthy controls. However, these results are contrary to two previous studies that indicate an inverse association between S-25(OH)D and ICV in healthy female students^22^ and in older adults with memory complaints^23^. The discrepancy in the findings could be due to nonlinearity; The two previous studies had several participants with S-25(OH)D > 100 nmol/l, while the current study only had three above 100 nmol/l. In the scientific literature, there are indications of a nonlinear relation between neonatal S-25(OH)D and the risk for schizophrenia^13^ and between S-25(OH)D and other disease outcomes^36^. Thus, it is not unlikely that the S-25(OH)D association could be nonlinear and that this nonlinearity explains the discrepancy in the findings. Due to few study participants with higher S-25(OH)D measures nonlinearity was not investigated in this study.

Prenatal or childhood S-25(OH)D measures were not available for study. Adult measurements could, considering the genetic influence on S-25(OH)D^35,36^, hypothetically reflect premorbid S-25(OH)D with potential effect on the brain and cranial development and/or disease risk as detected in the adult. Developmental vitamin D levels^13^ have been associated with increased risk for schizophrenia and animal models indicate altered gene expression following developmental vitamin D deficiency^20,21^. Thus, it is plausible that altered gene expression also occurs in humans with developmental vitamin D deficiencies. One could speculate that induced gene expression changes from micronutrient deficiency could influence the development of both the brain and cranium. Hypothetically, this could also be a precursor to the development of severe mental disorders although the data in this study does not support a significant patient effect on ICV. Such hypotheses warrant further investigation e.g. through studying neonatal S-25(OH)D or related gene expressions for morphological brain changes in childhood, adolescence or adulthood.

Although we have interpreted the observed association between S-25(OH)D and ICV along a genetic pathway, other factors beyond the genetic realm could also explain the association. It is possible that the observed association between S-25(OH)D and ICV is due to other factors not accounted for and that these factors are correlated with both S-25(OH)D and ICV in adults. Potentially, the association between S-25(OH)D and ICV could be mediated by lifestyle factors such as a sedentary indoors lifestyle, socioeconomic status, or education attainment. For instance, obesity in adolescence and adulthood have been associated with negative structural brain changes^41^. A recent meta-analysis reported that obesity was associated with higher prevalence of S-25(OH)D deficiency at all ages^58^. Thus, factors such as obesity could influence the cranium’s development, in addition to being associated with deficiencies. However, we did not observe an association between weight and S-25(OH)D levels although weight was found negatively associated with ICV. Lower socioeconomic status, a reported risk factor for schizophrenia^46^, has also been shown to influence brain structure in children negatively^45^, which in turn could influence the development of the cranium. These and other lifestyle related factors could have the potential to influence, or be influenced by, brain development and ICV as measured in the adult.

Unexpectedly, we also found a positive association between S-folate and S-25(OH)D levels which, to our knowledge, has not been previously reported. One possible explanation is through gastrointestinal health. There are indications that S-25(OH)D may influence intestinal permeability^59^. Vitamin D receptors are important for intestinal health^60^, their genetic expression has been associated with microbiome diversity^61^ and have together with 1,25-dihydroxyvitamin D been associated with increased intestinal folate absorption in *ex-vivo* rats^62^. Folate is synthetized by the intestinal microbiome^24^ and in one study folate was associated with intestinal vitamin D metabolism in mice^63^. Thus, S-25(OH)D could potentially influence S-folate, or vice versa, positively through improved functioning of the intestinal microbiome and/or absorption. Replication is warranted as it could also be a spurious finding or related to diet.

### Limitations

There are some limitations with the current study. The cross-sectional design makes it difficult to distinguish cause from effect. The patient group was heterogeneous, but limited sample size discouraged subgrouping. For the patients, serum samples were from fasting morning blood, while this was not necessarily true for controls, which could influence the results as S-folate increase with food intake^64^. The ethnic background of patients and controls differed with a higher proportion of non-Caucasians among patients.

In a later study, genetic associations and genetic regulation of S-25(OH)D on ICV should be investigated. For the current study sample, it is a limitation that we do not have adequate information on socioeconomic data, parental education of controls, diet or other lifestyle factors such as sun-exposure and outdoor physical activity. These factors could be associated with S-25(OH)D and ICV and this should be explored in future studies.

## Conclusion

We here report a significant positive association between adult S-25(OH)D and ICV. S-folate was trend-significantly associated with ICV. In addition, we report a positive association between S-folate and S-25(OH)D. S-25(OH)D was, through its significant association with S-folate, interpreted to explain the trend level S-folate association with ICV. We did not find significant differences between patients with severe mental disorders and healthy controls for ICV or serum measures. The results could be related to genetic regulation of S-25(OH)D, but the associations could also be mediated by other factors. Further studies are warranted for replication in larger samples and to investigate possible mechanisms and environmental influences, as well as implications for health and severe mental disorders.

## Electronic supplementary material

Supplemental Information
