# Supplementary material for: Vitamin D, Folate and the Intracranial Volume in Schizophrenia and Bipolar Disorder and Healthy Controls
Source: Sci Rep. 2018 Jul 17;8:10817. doi: 10.1038/s41598-018-29141-y (PMC6050333; doi:10.1038/s41598-018-29141-y)
Supplement: Supplementary file 1 — Supplemental Information [file 41598_2018_29141_MOESM1_ESM.pdf]

Supplemental Information

Title: Vitamin D, Folate and the Intracranial Volume in Schizophrenia and Bipolar Disorder and Healthy Controls.

Authors: Tiril P. Gurholt[a,b,*], Kåre Osnes[b], Mari Nerhus[a,c], Kjetil N. Jørgensen[a,b], Vera Lonning[a,b], Akiah O. Berg[a,d], Ole A. Andreassen[a,d], Ingrid Melle[a,d], Ingrid Agartz[a,b,e]

[a] Norwegian Centre for Mental Disorders Research (NORMENT), KG Jebsen Centre for Psychosis Research, Division of Mental Health and Addiction, Institute of Clinical Medicine, University of Oslo, Oslo, Norway.

[b] Department of Psychiatric Research, Diakonhjemmet Hospital, Oslo, Norway.

[c] Division of Mental Health, Akershus University Hospital, Lørenskog, Norway

[d] Division of Mental Health and Addiction, Oslo University Hospital, Oslo, Norway.

[e] Department of Clinical Neuroscience, Centre for Psychiatric Research, Karolinska Institutet, Stockholm, Sweden

Supplemental Information

Note 1

Supplemental information Table S1 presents the influence of ethnicity on demographic variables for patients. Briefly, there are 67 Caucasian (58.2% women) and 23 non-Caucasian patients (34.8% women), which gives a trend significant sex difference. Non-Caucasian patients have a heterogeneous ethnic background. Caucasian patients have a significantly earlier age of onset when compared to non-Caucasian patients (p=0.034). At MRI, there is a significant difference (p=0.0003) in average duration of illness; it is shorter non-Caucasian patients. S-25(OH)D (p=0.003) and S-folate (p=0.030) also differ significantly; On average, Caucasian patients have suboptimal and non-Caucasian patients insufficient S-25(OH)D. Non-Caucasian patients have lower S-folate than Caucasian patients, although within normal range. Neither the patients' education level, nor the parental education level, differ significantly based on ethnic background. There are no differences for the other demographic and clinical variables. The average number of days between MRI and blood sampling was 8.8±8.7 (mean ± standard deviation) for Caucasian and 5.5±6.2 for non-Caucasian patients.

The uneven number of Caucasian (N=67) and non-Caucasian (N=23) patients might influence the test reliability.

Supplemental Information

Table S1: Demographic and clinical characteristics for Caucasian and non-Caucasian patients with severe mental disorders.

	**Caucasian Patients (N=67)**	**Non-Caucasian Patients (N=23)**	**Wilcoxon rank sum test[1] or χ^2**	**p-value**
**Clinical information[2]**				
Women, N (%)	39 (58.2)	8 (34.8)	2.89	.089
Age (years)	31.4 (11.7)	28.3 (7.3)	.56	.579
Assessed summer, N (%)	29 (43.3)	11 (47.8)	.02	.893
Education (years)[3]	13.4 (2.4)	12.9 (2.1)	.91	.363
Mother college educated, N (%)[4]	40 (60.6)	8 (44.4)	.92	.337
Father college educated, N (%)[5]	36 (56.3)	9 (52.9)	0	1
Height (cm)	174.6 (9.2)	173.7 (8.4)	.02	.982
Weight (kg)	78.8 (18.8)	77.5 (16.5)	.10	.919
BMI	25.8 (5.4)	25.5 (3.9)	.11	.912
AAO (years)	21.4 (8.5)	24.6 (7.1)	-2.13	**.034**
DOI at MRI (years)	10.0 (8.9)	3.8 (3.9)	3.58	**.0003**
**Clinical tests**				
GAF-F	51.4 (12.3)	50.9 (12.2)	.41	.680
GAF-S	51.9 (13.3)	53.7 (13.4)	-.16	.871
Total PANSS score	49.0 (12.6)	51.7 (11.7)	-1.14	.255
Negative PANSS score	11.1 (3.6)	13.0 (5.8)	-1.05	.295
Positive PANSS score	11.3 (4.5)	10.8 (3.7)	.13	.900
**Serum measurements**				
S-25(OH)D (nmol/l)	52.8 (21.8)	37.2 (19.0)	3.00	**.003**
S-folate (nmol/l)	21.9 (10.1)	16.8 (6.8)	2.17	**.030**
**Imaging information**				
Estimated ICV (ml)	1481.0 (133.9)	1428.6 (107.3)	1.37	.171

Notes: Significance threshold p <0.05 indicated in bold. Two-sided Wilcoxon rank sum test applied for continuous variables and χ^2-test for categorical data. Patients: patients with severe mental disorders, MRI: magnetic resonance imaging, BMI: Body-Mass Index (weight in kg/meter squared), winter: November-April, summer: May-October, AAO: Age at onset, DOI: Duration of Illness, GAF: Global Assessment of Functioning, GAF-F: GAF–function scale, GAF-S: GAF-symptom scale, PANSS: Positive and Negative Syndrome Scale, S-25(OH)D: 25-hydroxyvitamin D, ICV: Intracranial Volume. [1] All continuous variables had a non-normal distribution by the Shapiro-Wilk normality test or from visual inspection of the variables histogram. [2] Presented as mean (standard deviation) if not stated otherwise.[3] Computation based on 55 (82.1%) of Caucasian patients and 23 (100%) of non-Caucasian patients.[4] Computation based on 66 (98.5%) Caucasian mothers and 18 (78.3%) non-Caucasian mothers. [5] Computation based on 64 (95.5%) Caucasian fathers and 17 (73.9%) non-Caucasian fathers.

Supplemental Information

Note 2

In Supplementary Table S2, for exploratory purposes we investigate the potential interactions between the serum measures and patient status for Model 1-3. The results show no significant associations between S-25(OH)D, S-folate and ICV limited to patients. Furthermore, given the significant contribution of assessment season on S-25(OH)D in Table 3, in Supplementary Table S3 we investigate the potential of an interaction between the serum measures and assessment season on ICV. There are no interaction effects.

In Supplementary Table S4, Model 1-3 and the post hoc models are compared using F statistics with the simpler model as the null hypothesis[1]. Briefly, the F statistics indicate that for Model 1 the inclusion of S-folate as a covariate in Model 3 yields a nonsignificant contribution (F=1.43, p=.233), while for Model 2 the inclusion of S-25(OH)D in Model 3 yields a significant contribution (F=6.62, p=.011). The inclusion of the interaction between the serum measures and patient status in the models does not give a significant contribution, nor does the inclusion of the interactions between the serum measures and assessment season.

Supplementary Table S5 assesses the potential interaction between S-folate and patient status, and between S-folate and assessment season, on S-25(OH)D for post hoc Model A. There is no significant patient specific or season specific associations for S-folate on S-25(OH)D. Model comparison revealed that the inclusion of the interaction terms does not give a significant contribution with (F=0.34, p=0.559) and (F=0.55, p=0.459), respectively.

Supplemental Information

Table S2: The association between S-25(OH)D, S-folate and ICV for patients and controls, including interaction between serum measures and group.

	**Dependent variable: ICV**								
	**Model 1 w/ group interaction**			**Model 2 w/ group interaction**			**Model 3 w/ group interaction**		
**Covariates**	**β**	**95% CI**	**p-value**	**β**	**95% CI**	**p-value**	**β**	**95% CI**	**p-value**
S-25(OH)D	.2	(.001,.4)	**.049**				.2	(-.02, .4)	.073
S-folate				.1	(-.1, .3)	.307	.1	(-.1, .3)	.514
Group[1]	.01	(-.2, .3)	.930	-.03	(-.3, .2)	.848	-.01	(-.3, .2)	.923
Age	-.00	(-.1, .1)	.992	-.00	(-.1, .1)	.978	-.02	(-.1, .1)	.807
Sex[2]	.8	(.5, 1.2)	**7.6e-06**	.8	(.4, 1.1)	**1.6e-05**	.8	(.5, 1.2)	**7.2e-06**
A. season[3]	-.04	(-.3, .2)	.724	.1	(-.2, .3)	.681	-.03	(-.3, .2)	.802
Weight	-.2	(-.4, -.1)	**.004**	-.2	(-.4, -.1)	**.007**	-.2	(-.4, -.1)	**.008**
Height	.4	(.2, .6)	**4.7e-05**	.4	(.2, .6)	**5.6e-05**	.4	(.2, .6)	**6.2e-05**
Ethnicity[4]	-.4	(-.8, -.001)	**.050**	-.5	(-.8, -.1)	**.020**	-.4	(-.8, .02)	.065
S-25(OH)D-by-Group[1]	.02	(-.2, .3)	.852				.00	(-.2, .3)	.974
S-folate-by-Group[1]				.03	(-.2, .3)	.790	.02	(-.2, .3)	.888

Notes: Regression model with N=181 participants (90 patients, 91 controls) and standardized continuous covariates. Model 1: S-25(OH)D on ICV, Model 2: S-folate on ICV, Model 3: S-25(OH)D and S-folate on ICV Significance threshold p<0.05 indicated in bold. Group: Patient-Control status, winter: November-April, summer: May-October, Ethnicity: Caucasian or non-Caucasian origin, CI: Confidence Interval, A. season: Assessment season. [1] Reference group: controls. [2] Reference sex: women. [3] Reference season: winter.
[4] Reference ethnicity: Caucasian.

Supplemental Information

Table S3: The association between S-25(OH)D, S-folate and ICV for patients and controls, including interaction between serum measures and assessment season.

	**Dependent variable: ICV**								
	**Model 1 w/ assessment season interaction**			**Model 2 w/ assessment season interaction**			**Model 3 w/ assessment season interaction**		
**Covariates**	**β**	**95% CI**	**p-value**	**β**	**95% CI**	**p-value**	**β**	**95% CI**	**p-value**
S-25(OH)D	.2	(.04, .4)	**.017**				.2	(.0003, .3)	**.050**
S-folate				.2	(-.01, .3)	.068	.1	(-.1, .3)	.169
Group[1]	.01	(-.2, .3)	.924	-.03	(-.3, .2)	.795	-.02	(-.3, .2)	.852
Age	-.00	(-.1, .1)	.993	-.00	(-.1, .1)	.974	-.02	(-.1, .1)	.804
Sex[2]	.8	(.5, 1.2)	**6.7e-06**	.8	(.4, 1.1)	**2.2e-05**	.8	(.5, 1.2)	**1.2e-05**
A. season[3]	-.04	(-.3, .2)	.719	.05	(-.2, .3)	.682	-.03	(-.3, .2)	.794
Weight	-.2	(-.4, -.1)	**.004**	-.2	(-.4, -.1)	**.010**	-.2	(-.3, -.04)	**.013**
Height	.4	(.2, .6)	**5.0e-05**	.4	(.2, .6)	**5.2e-05**	.4	(.2, .6)	**5.5e-05**
Ethnicity[4]	-.4	(-.8, -.02)	**.041**	-.4	(-.8, -.1)	**.026**	-.3	(-.7, .1)	.086
S-25(OH)D-by-A.season[3]	-.03	(-.3, .2)	.817				-.00	(-.3, .3)	.984
S-folate-by-A. season[3]				-.1	(-.3, .2)	.518	-.1	(-.5,.2)	.457

Notes: Regression model with N=181 participants (90 patients, 91 controls) and standardized continuous covariates. Model 1: S-25(OH)D on ICV, Model 2: S-folate on ICV, Model 3: S-25(OH)D and S-folate on ICV Significance threshold p<0.05 indicated in bold. Group: Patient-Control status, winter: November-April, summer: May-October, Ethnicity: Caucasian or non-Caucasian origin, CI: Confidence Interval, A. season: Assessment season. [1] Reference group: controls. [2] Reference sex: women. [3] Reference season: winter. [4] Reference ethnicity: Caucasian.

Supplemental Information

Table S4: Model 1-3 with/without interaction compared using F statistics.

	Initial Model		
**Comparing model**	**Model 1**	**Model 2**	**Model 3**
Model 3	F=1.43, p=.233	F=6.62, p=**.011**	
Model 1 w/ group interaction[1]	F=.04, p=.852		
Model 2 w/ group interaction[2]		F=.07, p=.790	
Model 3 w/ group interaction[3]			F=.01, p=.987
Model 1 w/ assessment season interaction[4]	F=.05, p=.817		
Model 2 w/ assessment season interaction[5]		F=.42, p=.518	
Model 3 w/ assessment season interaction[6]			F=.32, p=.724

Notes: Reference group: Controls. Interactions are between serum measures and Group. Model 1: S-25(OH)D on ICV, Model 2: S-folate on ICV, Model 3: S-25(OH)D and S-folate on ICV. Significance threshold $p<0.05$ indicated in bold. [1] Interaction: S-25(OH)D-by-group. [2] Interaction: S-folate-by-group. [3] Interactions: S-25(OH)D-by-group and S-folate-by-group. [4] Interaction: S-25(OH)D-by-Assessment season. [5] Interaction: S-folate-by-Assessment season. [6] Interaction: S-25(OH)D-by-Assessment season and S-folate-by-Assessment season.

Table S5: The association between S-folate and S-25(OH)D for patients and controls when including S-folate-by-group interaction or S-folate-by-assessment season interaction.

	**Dependent variable S-25-(OH)D**					
	**Post hoc Model A w/ group interaction**			**Post hoc Model A w/ A. season interaction**		
**Covariates**	**β**	**95% CI**	**p-value**	**β**	**95% CI**	**p-value**
S-Folate	.2	(.001, .4)	**.049**	.2	(.04, .4)	**.021**
Group[1]	-.1	(-.4, .2)	.546	-.1	(-.4, .2)	.598
Age	.1	(-.1, .2)	.293	.1	(-.1, .2)	.322
Sex[2]	-.1	(-.4, .1)	.327	-.1	(-.4, .1)	.347
A. season[3]	.5	(.2, .8)	**.001**	.5	(.2, .7)	**.001**
Ethnicity[4]	-.5	(-1.0, -.1)	**.015**	-.6	(-1.0, -.1)	**.009**
S-folate-by-Group[1]	.1	(-.2, .4)	.551			
S-folate-by-A. season[3]				.1	(-.2, .4)	.502

Notes: Regression model with N=181 participants (90 patients, 91 controls) and standardized continuous covariates. Post hoc Model A: S-folate on S-25(OH)D. Significance threshold $p<0.05$ indicated in bold. Group: Patient-Control status, winter: November-April, summer: May-October, Ethnicity: Caucasian or non-Caucasian origin, CI: Confidence Interval, A. season: Assessment season. [1] Reference group: controls. [2] Reference sex: women. [3] Reference season: winter. [4] Reference ethnicity: Caucasian.

Supplemental Information

References

1 Hastie, T., Tibshirani, R. & Friedman, J. *The Elements of Statistical Learning: Data Mining, Inference, and Prediction, Second Edition*. (Springer New York, 2009).
